# Intra-articular synovial lipoma of the knee joint

**DOI:** 10.1259/bjrcr.20150061

**Published:** 2015-05-26

**Authors:** L Poorteman, H Declercq, P Natens, K Wetzels, F Vanhoenacker

**Affiliations:** ^1^Department of Radiology, AZ Sint Blasius, Dendermonde, KU Leuven, Belgium; ^2^Department of Orthopedics, AZ Sint Blasius, Dendermonde, Belgium; ^3^Department of Pathology, AZ Sint Blasius, Dendermonde, Belgium; ^4^Department of Radiology, AZ Sint-Maarten, Duffel-Mechelen, University Hospital Antwerp, University of Ghent, Belgium

## Abstract

We present a rare case of an intra-articular synovial lipoma, which was diagnosed in a patient after a knee trauma. MRI is the imaging modality of choice to suggest the diagnosis preoperatively, by demonstrating a well-delineated fat-containing lesion. The differential diagnosis of an intra-articular lipomatous lesion consists of lipoma arborescens and synovial lipoma.

## Clinical presentation

A 72-year-old female presented to the orthopaedic surgeon after a bike accident. There was joint effusion and pain over the medial but mostly the lateral joint space of the right knee. No masses were palpable. Further medical history of the patient was unremarkable.

## Investigations and imaging findings

**Figure 1.**
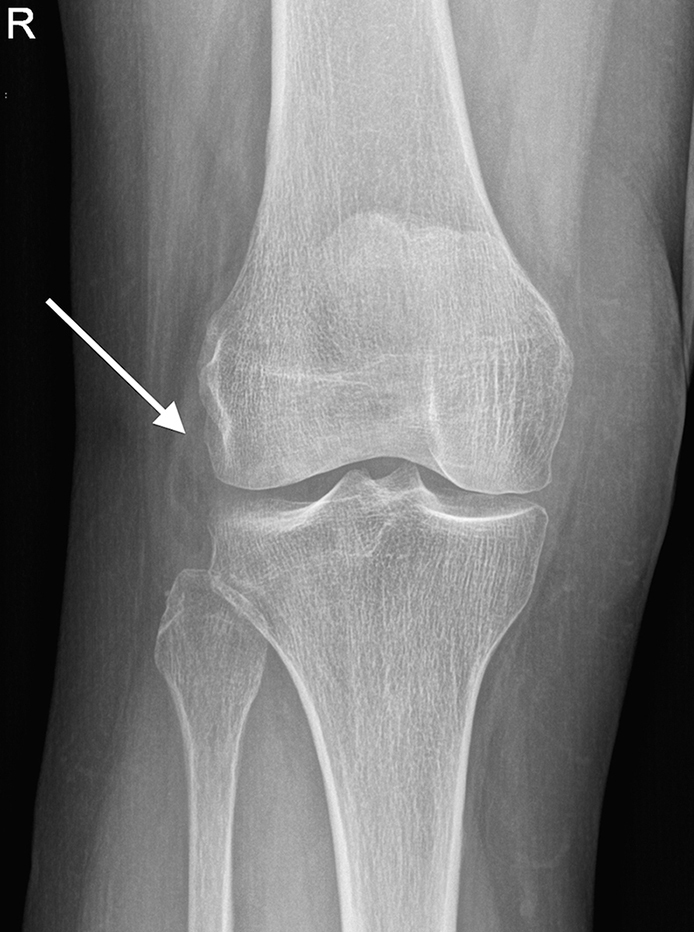
Anteroposterior radiograph of the right knee shows a subtle low-density structure adjacent to the lateral joint space, suggestive of a fat-containing lesion (arrow).

**Figure 2.**
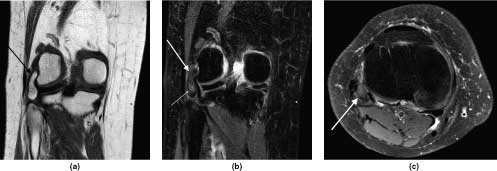
MRI images of the right knee. (a) *T*_1_ weighted coronal image showing a lipomatous mass in the lateral compartment of the knee joint, located between the lateral collateral ligament and the popliteus tendon (black arrow). (b,c) Fat suppressed *T*_2_ weighted coronal and axial image showing the lipomatous mass (large white arrows) with subtle signs of inflammation (small white arrow in b).

Radiographs were initially interpreted as normal (Figure 1). Retrospectively, a subtle low-density structure adjacent to the lateral joint space can be seen, suggestive of a fat-containing lesion. There were no soft tissue calcifications. MRI showed a tear on the body and posterior horn of the medial meniscus. In addition, a well-circumscribed soft-tissue lesion with fatty consistency was seen underneath the lateral collateral ligament and the popliteus tendon. The mass was slightly inhomogeneous, with some subtle intralesional foci of intermediate signal intensity on *T*_1_ weighted images and high signal intensity on *T*_2_ weighted images (Figure 2).

## Differential diagnosis

The diagnosis of an intra-articular lipomatous lesion was made based on these imaging features. Although there is an extensive range of intra-articular masses (Table 1),^1^ the differential diagnosis in our case could be limited to fat-containing lesions. Our preferential imaging diagnosis was synovial lipoma. Because the patient’s complaints and pain were predominantly located at the lateral joint space, the lesion could be considered as the main cause of her knee pain. No biopsy was performed preoperatively.

**Table:** Table 1. Classification of intra-articular masses. Adapted from Sheldon et al^1^

Intra-articular masses
Non-infectious synovial proliferative processes Lipoma arborescens Synovial lipoma Synovial osteochondromatosis Pigmented villonodular synovitis Rheumatoid arthritis
Infectious granulomatous diseases Tuberculous arthritis Coccidioidomycosis arthritis
Deposition diseases Gout Amyloid arthropathy
Malignancies Synovial chondrosarcoma Synovial metastases
Vascular malformations Arteriovenous malformations
Vascular tumours Synovial haemangioma
Miscellaneous conditions Cyclops lesion

## Treatment

**Figure 3.**
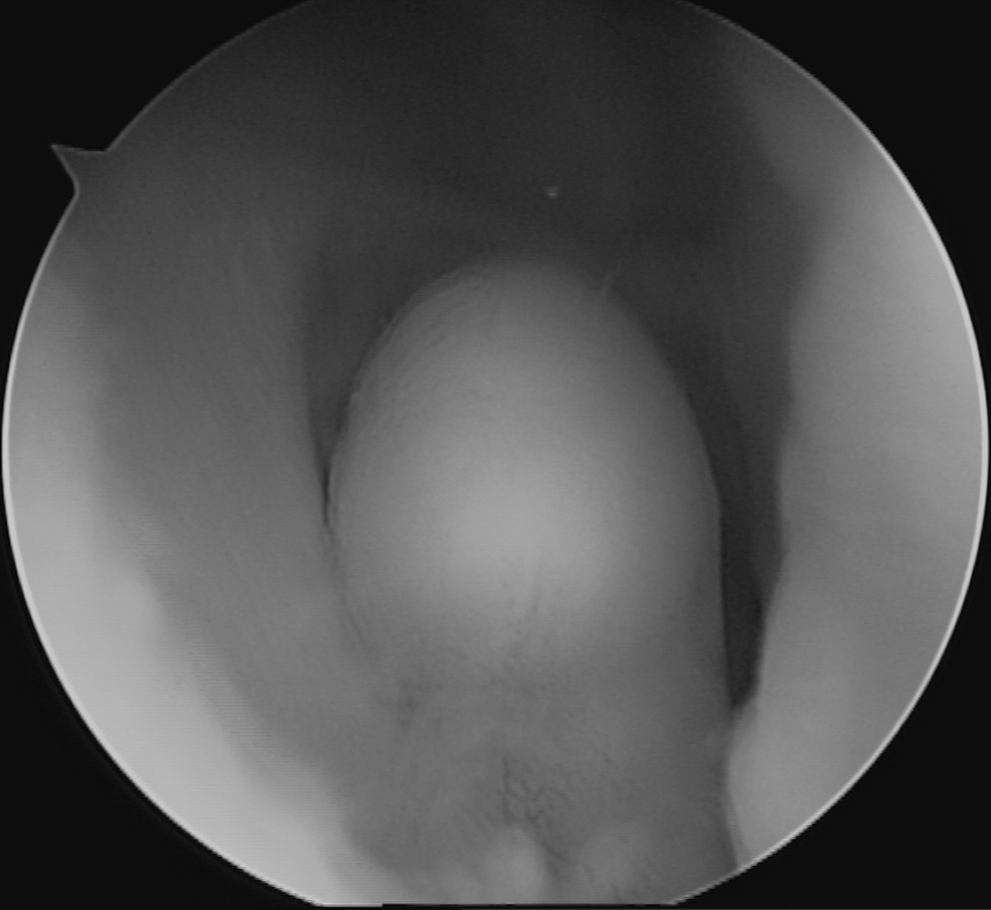
Arthroscopic image showing the intra-articular, smooth-bordered lipomatous mass in the lateral recess of the knee joint.

Arthroscopy confirmed a medial meniscus tear as well as a well-delineated synovial-based mass at the lateral joint recess. The white to yellowish aspect of the mass was indicative of a lesion of fatty consistency. There was subtle vascular engorgement on the surface of the lesion (Figure 3). The mass was excised and the resected specimen was referred for histopathological examination.

## Outcome and follow-up

**Figure 4.**
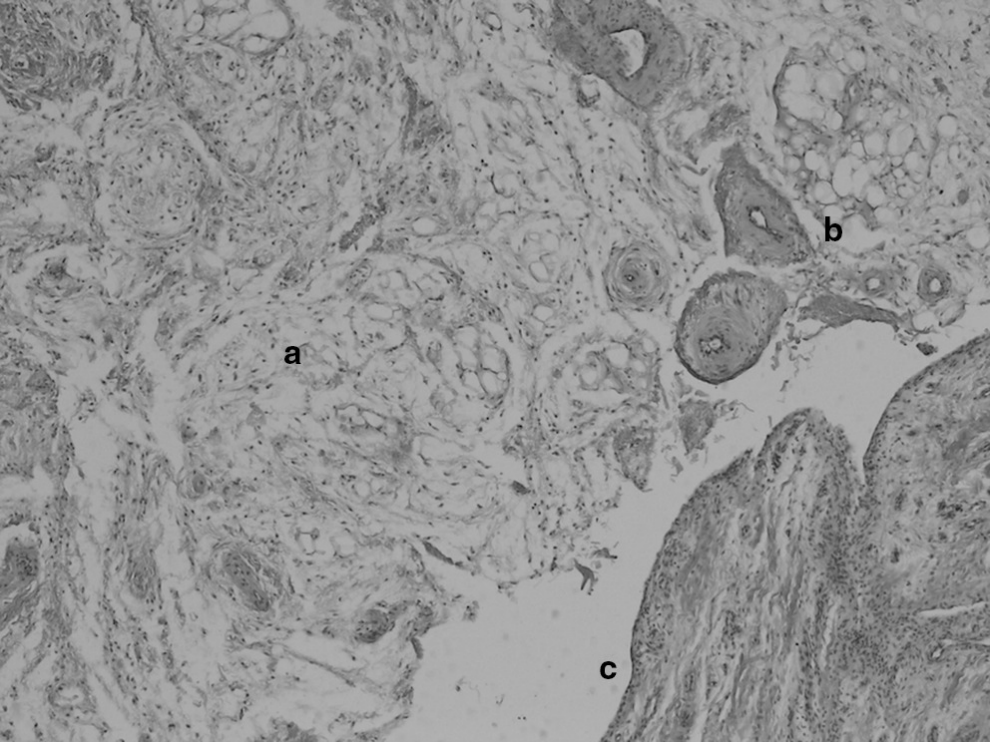
Microphotograph of the obtained sample showing the presence of normal fat cells and surrounding connective tissue (a) and feeding arterial vessels (b), together with normal synovial-lining cells (c). The synovium looks detached from the underlying fat cells owing to the preparation of the histological sample.

Histopathological examination revealed normal fibroadipose and synovial tissue (Figure 4), which confirmed the diagnosis of a synovial lipoma.

The post-operative course was uneventful and the patient left the hospital after 2 days.

## Discussion

Fat-containing intra-articular soft-tissue masses are rare and include lipoma arborescens and the even more rare synovial lipoma. Although lipomas are common benign neoplasms that occur almost anywhere in the body, intra-articular occurrence is very rare.^2^ An intra-articular lipoma is a solitary lipomatous lesion with a thin fibrous capsule and a vascular pedicle, often covered by synovial tissue. Since the first case report in 1979,^3^ we found only 21 case reports in the Anglo-Saxon literature describing this type of lipoma, of which almost all are located in the knee joint (18 cases),^3–20^ 1 in the hip joint,^21^ 1 in the lumbar spine^22^ and 1 in the tarsometatarsal joint.^23^ One of these reports^14^ describes the lesion at the lateral recess of the knee joint, very similar to the location in our patient. A synovial lipoma is supposed to originate within the joint, either by penetrating the synovial membrane or by fatty overgrowth from the intra-articular synovial tissue. It can cause locking of the knee when the lipoma gets impinged between the articular surfaces, within the intercondylar notch or upon the meniscus, or it can even get strangulated if it twists around its stalk, causing sudden knee pain.^14^ It has been suggested that a synovial lipoma occurs *de novo,* unrelated to trauma.^13^ We postulate that, in our case, the mass was pre-existent, but could have become inflamed owing to trauma, resulting in pain at the lateral joint space. The signal intensity of the lesion is similar to fat on all MRI sequences. On radiographs, a low-density structure may be present.^13^ Treatment of small lesions consists of arthroscopic resection, but larger lesions may require resection by open surgery.^13^

The most important differential diagnosis of a synovial lipoma is lipoma arborescens. This condition is caused by villous synovial proliferation with hyperplasia of the subsynovial adipose tissue.^1^ The exact aetiology is unknown, but unusual response to chronic synovial irritation or trauma have been suggested as possible causes leading to this condition. It most commonly occurs in the suprapatellar bursa of the knee joint and causes a painless, slowly progressive swelling of the joint with recurrent effusions. As in synovial lipoma, this lesion has a fatty density on radiographs.^1,2^ On ultrasound, a hyperechoic frond-like mass can be seen with wave-like motion within the intra-articular effusion.^1^ MRI investigation shows synovial masses with signal intensity similar to fat on all pulse sequences.^1,2^ Microscopically, the histological characteristics of the lipoma arborescens are similar to synovial lipoma. The differential diagnosis is mainly made based on the macroscopic appearance.^14^ The treatment consists of synovectomy,^1,2^ with a low recurrence rate.^1^

Other lesions involving the synovium of articulations are listed in Table 1,^1^ but further discussion of these rare lesions is beyond the scope of this report.

## Learning points

1. Synovial lipoma is a rare intra-articular lesion.
2. The lesion is usually located within the knee joint.
3. MRI is the preferred imaging technique for diagnosis.
4. The most significant differential diagnosis is lipoma arborescens, which has a different macroscopic appearance.
5. Treatment consists of (arthroscopic) resection.
